# The Mediating Effect of Innovation in Between Strategic Orientation and Enterprise Performance: Evidence From Malaysian Manufacturing Small-to-Medium-Sized Enterprises

**DOI:** 10.3389/fpsyg.2022.887895

**Published:** 2022-05-11

**Authors:** Abdullah Al Mamun, Naeem Hayat, Syed Ali Fazal, Anas A. Salameh, Noor Raihani Zainol, Zafir Khan Mohamed Makhbul

**Affiliations:** ^1^UKM-Graduate School of Business, Universiti Kebangsaan Malaysia, Bangi, Malaysia; ^2^Global Entrepreneurship Research and Innovation Centre, Universiti Malaysia Kelantan, Kota Bharu, Malaysia; ^3^Faculty of Business Administration, University of Science and Technology Chittagong, Chittagong, Bangladesh; ^4^Department of Management Information Systems, College of Business Administration, Prince Sattam Bin Abdulaziz University, Al-Kharj, Saudi Arabia

**Keywords:** market orientation, customer orientation, entrepreneurial orientation, product and process innovation, performance of manufacturing SMEs

## Abstract

Strategic orientation and innovation are vital determinants for accelerating the performance of small-to-medium-sized enterprises (SMEs). However, there is a lack of empirical evidence confirming the innovation at the product and process levels that instigated the SMEs’ performance. Moreover, the mediating effect of process and product innovation can play a significant role in strategic orientation and manufacturing SMEs’ performance. In this respect, this study aims to examine the mediating effect of product and process innovation between strategic orientation (i.e., market, entrepreneurial, and customer orientation) and the performance of Malaysian manufacturing SMEs. The questionnaire survey gathered data from 360 manufacturing SMEs and was analyzed using partial least square structural equation modeling (PLS-SEM) to achieve these research objectives. The study analysis established that customer and entrepreneurial orientation significantly influence product and service innovation. However, the market orientation is significant for process innovation but insignificant for product-level innovation among SMEs. The study’s consequences exposed that process innovation has significantly mediated between the strategic (market, customer, and entrepreneurial) orientation and SMEs performance. It implies that market, entrepreneurial, and customer-related strategies would substantially improve SMEs’ performance by harnessing innovation at product and process levels. The core insights provided by the current work are to strengthen the strategic orientation that can promote product and process innovation, thereby harnessing the SMEs’ performance. Additionally, the study’s significance and limitations were reported at the end.

## Introduction

Small-to-medium-sized enterprises (SMEs) substantially contribute to a country’s economic development by creating multiple jobs, efficiently using resources, increasing national income, and reducing poverty (Behnam and Cagliano, 2019; Yousaf et al., 2021). As the backbone of the Asia-Pacific economy, SMEs employ portions of the total workforce, accounting for more than 97% of all enterprises (Nolan, 2017). SMEs development is the main driving force of Malaysian national economic growth, leading them to be a developed nation by 2030 (Nasir et al., 2017). However, strategic direction and innovation are the prerequisite to accelerating the performance of SMEs (Cozzarin, 2017). Strategic orientation reflects the processes, principles, applications, and decision-making techniques that guide the organization’s activities. It responds to the external environment and generates the intended behavior to improve performance and organizational sustainability (Fernando et al., 2019; Robb and Stephens, 2021; Schmidt et al., 2021).

In addition, innovation is a crucial element for any nation’s economic progression that creates competitiveness among industries and substantially influences the firm performance and broadly the economic growth of the nation (Adedeji et al., 2017; Rasheed et al., 2020). Innovation leads to superior profitability and competitive advantage that can create new wealth directly and indirectly by altering and enhancing existing resources (Tutar et al., 2015; Pulka et al., 2021). It also enables companies to enhance product quality and services by differentiating themselves from their competition, thus contributing to competitive advantage and sustainability (Siti et al., 2017; Muangmee et al., 2021). Cozzarin (2017) specified that innovation usually refers to a product or process-related innovation by default. Moreover, Girod and Whittington (2017) indicated that SMEs practicing product-related innovations performed better in total sales and exports.

Small-to-medium-sized enterprises contribute significantly to national growth and employment; SMEs’ performance is critical to national development (Kamalaldin et al., 2021). The emerging economies also need to concentrate on developing innovation among the SMEs to remain competitive and harness economic growth with the superior performance of the SMEs sector (Akhtar et al., 2021; Kiani et al., 2021). However, SMEs-level innovation requires the necessary strategic acumen and commitment to engage in product or service-level innovation (D’souza et al., 2021). We respond to the Nasir et al. (2017) call to explore the SMEs’ strategic orientation, nurturing innovation, and instigating the SMEs’ performance. Firm-level internal resources and capabilities guide product and process-level innovation (Cozzarin, 2017; Kamalaldin et al., 2021). We contribute to the existing literature by determining the firms’ level of strategic orientation and instigating product and process-level innovation while promoting the SMEs’ performance. Furthermore, evaluating the mediating effect of product and process innovation on the relationship between firms’ level of strategic orientation and SMEs’ performance.

In addition, the study addresses the inadequacy of several empirical proposals and explorations related to strategic direction and innovation (Wang et al., 2016). Particularly from the perspective of Malaysian SMEs, the study is essential since only 21–42% of Malaysian-based firms are considered innovative (SMEinfo, 2021). Manufacturing SMEs make substantial contributions to economic growth (Nolan, 2017). The manufacturing sector plays a crucial role in other industries (Department of Statistics Malaysia [DOSM], 2021). To support Malaysian manufacturing SMEs, the government provides support and assistance to 96.6% of all industrial organizations in the country (SMECrop, 2020). It was acknowledged that the manufacturing sectors maintained their dominant position as Malaysia’s main economic activities for SMEs and the GDP of Malaysia (Aziz et al., 2014). However, Malaysian SMEs shared in the national gross domestic product (GDP) and dropped slightly to 38.2% in 2021 from 38.9% in 2020 (Department of Statistics Malaysia [DOSM], 2021). The drop occurred due to COVID-19 and prolonged movement control orders (MCO) in Malaysia. However, the chemical, rubber, and petroleum sectors posted positive growth for the same period (Department of Statistics Malaysia [DOSM], 2021). Significant declines were observed in the construction, real estate, and mining sectors. The SMEs’ contribution to export performance improved and touched 69.3% of the SMEs’ production for 2020–2021.

The growth of manufacturing SMEs has become more competitive through various international cross-border trade agreements such as ASEAN, TPP, and RCEP. To date, manufacturing SMEs worldwide face stiff competition, thus actively developing effective strategies to compete with cheaper alternative products from countries such as India and China (Adedeji et al., 2019). Nevertheless, the weaknesses in these manufacturing sectors need to be addressed, such as low innovation investment, limited internationalization, and increased competition (Cozzarin, 2017). Adiguzel (2019) postulated that the success of manufacturing SMEs in increasingly competitive markets depends mainly on the extent to which SMEs engage in incorporating innovation in products and processes.

Although governments across the nation have formulated policies that consider SMEs’ ability to innovate, the very act of innovation remains a complex problem, especially for SMEs in emerging countries. In Malaysia, the Malaysian government runs 55 programs to infuse innovation and technology adoption to improve SMEs’ performance (SMEinfo, 2021). The shared prosperity vision 2030 advanced the development of SMEs’ innovation to achieve improved performance, even though only 21–42% of the local businesses are considered innovative (SMEinfo, 2021). In conjunction with the context, Klewitz (2017) found that strategy-oriented features, such as market orientation, enhanced the innovative nature of SMEs, leading to superior performance. Hence, the perception of the immense scope of the performance displayed by manufacturing SMEs and its close association with innovation and strategic orientation. Therefore, we examined the mediating role of product and process innovation in market, customer, entrepreneurial orientation, and corporate performance in Malaysian manufacturing SMEs.

Achieving innovation remains a driving force among SMEs to maintain a competitive market position and achieve economic performance. However, innovation is very narrowly defined as adopting a new idea or program. Innovation can be segregated into a product, process, and business model or management level of innovation (Rasheed et al., 2020). However, product innovation is known at the SMEs level, but scant literature is available on process innovation in SMEs. SMEs’ internal resources can play a significant role in activating firm-level innovation and superior firm performance. Therefore, the current study aims to explain the strategic orientation (market, customer, and entrepreneurial) prompting product and service-level innovation and empowering the SMEs’ performance among Malaysian SMEs.

## Literature Review

### Theoretical Stance

This work utilizes the resource-based view (RBV) to explain the firm’s internal resources instigating performance through innovation. Internal resources empower firms to gain a competitive advantage by developing capabilities for superior performance (Muangmee et al., 2021). Firm-level resources build the capacity to be innovative in the product and process (Pulka et al., 2021). It is good to explain the firm’s level of orientation instigating innovation aptitude at the product and process level for sustainable firm performance (Chatterjee et al., 2021). Market orientation allows one to understand the market and put forth the effort to remain competitive by making the appropriate product or process improvements (Akhtar et al., 2021). The customer orientation empowers the firms to work with the customers to comprehend and improve the product and process to meet the customer’s expectation for superior firm performance (Han and Zhang, 2021). Lastly, the entrepreneurial orientation facilitates the activation of internal resources to engage in firm resource utilization actively, leading to innovatively acting in a highly competitive market to attain performance (Muangmee et al., 2021).

### Firm Innovation, Product, and Process Innovation

Firm-level innovation becomes the source of competitive advantage for the firm (Chatterjee et al., 2021). The firm-level innovation deals with generating new ideas and using processes and technology to promote the firm’s competitive position in the market (Forcadell et al., 2021). Firms are engaged in developing new product ideas or improving existing products or services to gain a market-leading position (Chatterjee et al., 2021). Continuous innovation requires a sustainable strategy based on the firm dealing with innovation at the product and process level (Han and Zhang, 2021).

Product innovation describes the firm engaged in the release of new products and services to meet market demand in a dynamic marketplace (Murmura et al., 2021). The changing market environment drives the firm’s capabilities, leading to product-level innovation (Behnam and Cagliano, 2019). Product innovation relates to adding new attributes to the product and improving the product quality while meeting the continuously changing market demands (Chatterjee et al., 2021).

Process innovation involves using technology to change the production process or the innovative delivery of services (Kamalaldin et al., 2021). Process innovation is based on working with marketing intelligence and improving the product design and processes while promoting the firm’s level of competitiveness (Arboretti et al., 2021). Firm-level process innovation acts as the branding strategy and streamlines processes to bring effectiveness and efficiency, harnessing the firm’s competitive market position (Peters and Buijs, 2021).

However, using technology and innovation in products and processes requires dynamic capabilities at the firm level to instigate the right innovation (Chirumalla, 2021). Product and process innovation build the firm’s competitiveness and offer the firm the ability to remain competitive and achieve sustainability (Arboretti et al., 2021).

## Hypotheses Development

### Market Orientation and Innovation

Market orientation reflects both the goals and the company’s culture; it wants to remain focused on creating value for its customers to remain competitive (Siti et al., 2017). Obtaining, disseminating, and responding to market-generated information obtained from current and potential competitors (Nasir et al., 2017). Wang et al. (2016) pointed out that adopting technological innovations was crucial. It is why the existing literature insists too much on the association between market orientation and business innovation (Tutar et al., 2015). Innovative businesses are extremely market-driven, and they make every effort to stay competitive by developing new products or adding new features to their present ones to maintain a strong market position (Suriati, 2014). Being market-oriented means having a strategic adaptable mindset; businesses strive to recognize current market trends and must use technology and market-driven strategies to stay competitive in today’s highly competitive market (Schmidt et al., 2021).

Specifically, Na et al. (2019) revealed that market orientation positively improves product-related innovations in terms of product innovation. At the same time, concerning process innovation, Wang et al. (2016) argued that market orientation is associated with diverse forms of innovation, such as cultural, product, processes, and organizational cultural innovation. Since the concept of market orientation emphasizes the presentation of innovative products and/or services as a response to market information, it could be presumed as an innovative behavioral category (Cozzarin, 2017; Utami et al., 2022). Indeed, market orientation is depicted as constantly and proactively aiming to remain ahead of market competition and utilizing the firm’s resources to enhance overall innovation capacity and improve the firms’ innovative products or services offerings (Adiguzel, 2019). In addition, existing literature shows that market orientation positively influences products incorporating green innovation to mitigate climate issues (Wang et al., 2016). Recently, Akhtar et al. (2021) postulated that market orientation harnesses product and process-level innovation among Pakistani SMEs. Taking note of the above discussion, we would like to propose the following:

H1a: Market orientation significantly influences production innovation.
H1b: Market orientation significantly influences process innovation.

### Customer Orientation and Innovation

Customer orientation highlights placing consumers at the center of the strategic point to bring superior business performance (Wang et al., 2016). Nasir et al. (2017) have emphasized the need for enterprises to focus more on target customer segments (for example, shape different offers, messages, and services) and involve consumers at the individual level. Customer orientation enables companies to understand their market and thus develop complementary and product-related servicing plans to satisfy consumer demands (Wang et al., 2016). Khan and Khan (2019) further stated that customer orientation involves all activities to meet the needs and demands of present and potential consumers to achieve superior performance. A customer-focused firm indirectly affects innovation performance (Kim, 2017). Moreover, Akhtar et al. (2021) postulated that customer orientation influences, directly and indirectly, the innovation of products and services in manufacturing and servicing industries (Wang et al., 2016).

According to Salunke et al. (2019), customer orientation, especially in innovation-related projects, has positively impacted the success of new product-related development, with an increase in impact and its degree of innovation. However, extreme customer orientation negatively impacts innovation (Panagopoulos et al., 2017). Previous studies’ contradictory and inconclusive results indicate that the correlation between customer orientation and such innovation (product and process) requires further exploration. We like to propose the following:

H2a: Customer orientation significantly influences production innovation.
H2b: Customer orientation significantly influence process innovation.

### Entrepreneurial Orientation and Innovation

Entrepreneurial orientation reflects an organization’s tendency to explore newer market opportunities and strengthen its present market status (Aziz et al., 2014). Such orientation reflects a firm’s decisions, continuous incisiveness, and application, which allow new business-related opportunities to be created (Nasir et al., 2017). Entrepreneurial orientation is perceived as a blend of three constructs. First, innovativeness is involved encouraging and supporting innovative experimentation, ideas, and creativity likely to result in innovative services, products, or processes (Aziz et al., 2014). Second, risk-taking is concerned with the degree to which individuals differ from others in his/her willingness to take risks (Roxas et al., 2017). Third, pro-activeness, focusing on being the first mover and other actions to secure and protect market share (Aziz et al., 2014). Previous research has revealed that entrepreneurial orientation strengthens the rewards of knowledge-based resources and affects innovation performance utilizing knowledge management (Roxas et al., 2017).

Precisely, in terms of product innovation, Amankwah-Amoah et al. (2019) revealed positive effects of entrepreneurial orientation upon product-related innovativeness. On the other hand, Adedeji et al. (2019) found that strategically oriented enterprises are more involved in innovative firm processes. In general, taking risks, being a trendsetter for change, and innovating are unique attributes of entrepreneurial-oriented enterprises (Kiani et al., 2021). Muangmee et al. (2021) documented that entrepreneurial orientation promotes innovation among Thai SMEs.

Moreover, entrepreneurial orientation plays a pivotal role in innovation by promoting values of receptiveness that constitute innovativeness (Kiani et al., 2021). Being proactive about emerging opportunities and responding with innovative strategies are the fundamental requirements of being entrepreneurially oriented (Tutar et al., 2015). It facilitates a firm’s capability to discern the appropriate resources for integration, thus initiating innovation. Rasheed et al. (2020) established that SMEs’ top leadership entrepreneurial attitude effectively helps SMEs engage in the process level of innovation. Taking note of the above discussion, we would like to offer the following hypotheses:

H3a: Entrepreneurial orientation significantly influences product innovation.
H3b: Entrepreneurial orientation significantly influences process innovation.

### Innovation and Firm Performance

Innovation comprises creativity, new processes, research and development, new services and products, and innovative technologies (Cozzarin, 2017). Scholars are interested in the role played by innovation capability for acquiring superior performance (Tutar et al., 2015). The reconfiguration of firm resources empowers superior performance (Girod and Whittington, 2017). Indeed, innovation and organizational performance have a positive linkage (Behnam and Cagliano, 2019). For innovations to be efficient and ultimately successful, they must result in substantial change, preferably improvements in products, services, or processes compared with previous achievements (Nasir et al., 2017). Therefore, we perceived innovation as a process and product innovation. Process innovation is reflected as improved processes related to innovation and manufacturing design of new products based on added features (Suriati, 2014). Accordingly, Behnam and Cagliano (2019) posited that product innovations and process-related innovations positively influence market performance. Product innovation can further protect companies from market and competition turbulence and is one of the critical sources of superior market performance (Nasir et al., 2017). Similarly, process-level innovation empowers SMEs to gain a productivity-based cost advantage that yields efficiency and competitiveness for the firm (Rasheed et al., 2020). Process-level innovation helps SMEs achieve growth and business leadership positions. We like to propose the following:

H4a: Product innovation significantly influences enterprise performance.
H4b: Process innovation significantly influences enterprise performance.

### Mediating Effect of Innovation

In this study, market, customers, and entrepreneurial orientation were conceptualized as the main building blocks of innovation, while an association between innovation and business performance was also formulated. The study predicts that innovation plays a mediating role in the link between market, firm, and customer orientation and firms’ market performance among Malaysian manufacturing SMEs.

Kim (2017) explained that the strategic direction (including its dimensions) serves as a history of innovation characteristics at the firm level. The following function of this innovation is market performance, meaning an indirect effect of the strategic orientation and its dimensions on the company’s performance, innovation being a mediating factor. In a similar case, Tutar et al. (2015) have empirically described the indirect effect of the dimensions of strategic direction on firm performance, also influenced by service innovation.

HM1a: The relationship between market orientation and enterprise performance is significantly mediated by product innovation.
HM1b: The relationship between customer orientation and enterprise performance is significantly mediated by product innovation.
HM1c: The relationship between entrepreneurial orientation and enterprise performance is significantly mediated by product innovation.
HM2a: The relationship between market orientation and enterprise performance is significantly mediated by process innovation.
HM2b: The relationship between customer orientation and enterprise performance is significantly mediated by process innovation.
HM2c: The relationship between entrepreneurial orientation and enterprise performance is significantly mediated by process innovation.

**FIGURE 1:**
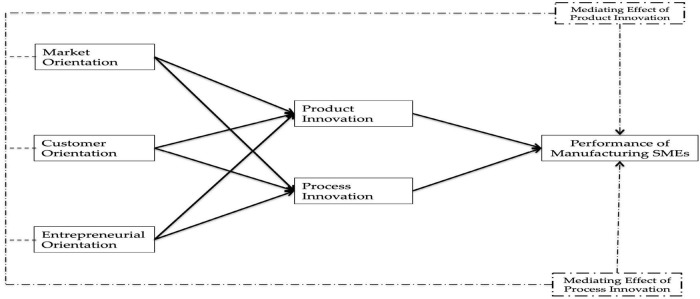
Research framework.

Based on the above critical literature review, the following proposed hypothesized model is developed and tested in this study (see Figure 1).

## Materials and Methods

### Sampling Technique and Sample Size

We used a cross-sectional approach to evaluate the influence of strategic focus on innovation and performance of manufacturing firms in Malaysia. Malaysian SMEs enlisted on the Malaysian Business Directory public website, provided by the SME Info Portal, formed our sampling frame (SMEinfo, 2021). SMEs owners and/or high-level managers in registered manufacturing firms make up the population frame. The 2020 Economic Census found that 58,718 firms exist in the Manufacturing SMEs category (SMECrop, 2020). From the sampling list, 400 SMEs were randomly selected from Johor, Selangor, Perak, Penang, Terengganu, and Kelantan to identify potential respondents. These states comprise the majority (79%) of manufacturing SMEs in Malaysia. From the selected 400 samples, 360 structured interviews were carried out with top-level managers. The data collection was performed from December 2020 to March 2021.

### Research Instrument Development

A survey questionnaire was employed for this study. To capture “market orientation,” 10 questions items were adapted from Deshpande and Farley (1998), 12 items from Ramani and Kumar (2008) for “customer orientation,” and six items for “entrepreneurial orientation” from Gonzalez-Benito et al. (2009), with minor contextual modifications. Similarly, based on the specific scope of the study, “Product innovation” was captured using seven items from Suriati (2014), while eight items were borrowed from Suriati (2014) to measure process innovation. Finally, we adapted subjective performance items from Siti et al. (2017) to capture “firm performance.” A standardized seven-point Likert scale (1 is “strongly disagree” and seven is “strongly agree”) was used for all variables.

### Common Method Variance

To gage the effect of common method bias and provide procedural remedies, we constructed the items carefully and informed the respondents that the responses would be evaluated anonymously and that there existed no wrong or right answers during the data collection procedure (Podsakoff et al., 2003). We assumed Harman’s (1976) one-factor test for statistical remedy, as Podsakoff et al. (2003) recommended. Finding that component one explains 42.6% of the variance, lower than the maximum threshold, 50%. Furthermore, a correlation of the latent constructs above 0.9 is considered an indicator of common method variance (Kock, 2015). The highest correlation between the constructs is 0.609 (entrepreneurial orientation and product innovation), indicating no severe common method variance (CMV) issue for the current study data (Kock, 2015).

### Data Normality

Following Peng and Lai (2012) for estimating models, we tested multivariate normality utilizing the online tool named “Web Power.” The test findings reported Mardia’s multivariate skewness, *p* < 0.05, which confirms non-normality in the dataset of this study. Moreover, the kurtosis coefficient for this model is 45.94, and the *p*-value is less than 0.05, which confirms the dataset’s non-normality.

### Data Analysis Technique

For data analysis, partial least squares structural equation modeling (PLS-SEM) was used with the Smart PLS software 3.2. PLS-SEM is a multivariate analysis instrument to gage the path models with composites’ latent constructs (Hair et al., 2019). Smart PLS 3.2 empowers the researcher to tackle small, non-normal datasets. Furthermore, SmartPLS 3.2 has a casual-predictive nature with an undisturbed supposition of goodness-of-fit estimation compared to the covariance-based SEM (Chin, 2010). Two-step techniques were used to analyze the data with PLS-SEM, and the first measurement was performed to test the model’s reliability and validity at the construct level (Hair et al., 2019). The second stage was executed to estimate the structural model and investigate study hypotheses with significance levels (Chin, 2010). Hair et al. (2019) performed model estimation with *r*^2^, *Q*^2^, and the effect size *f*^2^ describing the path effect from the exogenous construct to the endogenous construct. The current study had multivariate non-normality and an experimental design with a predictive nature. Therefore, we utilized the Smart PLS 3.2 to analyze the current study data.

## Results and Discussion

### Profile of the Selected Manufacturing Small-to-Medium-Sized Enterprises

Usable data were gathered from 360 Malaysian manufacturing SMEs, mostly established between 1988 and 2010. The mean time of operation was 19.49 years [standard deviation (SD) = 11.32 years], and the mean number of employees was 85.25 (SD = 57.66). In terms of types of enterprises, the data included basic metal firms (13.6%); chemicals, including petroleum (8.6%); electrical and electronics (11.4%); fabricated metal (6.1%); food, beverage, and tobacco (12.5%); machinery (5.8%); manufacture of furniture (5.6%); medical, precision, and optical instruments (2.2%); non-metallic mineral (1.9%); paper, printing, and publishing (5.6%); plastic (7.8%); recycling (5.0%); rubber (2.5%); textile, wearing apparel, and leather (1.7%); transport (0.8%); wood and other wood products (not excluding furniture) (3.9%); and others (5.0%). Lastly, most interviewees held a mid-level management position (53.9%), followed by top management (35.8), and owner/CEO (6.4%).

### Study Constructs Reliability and Validity

The sales orientation, customer orientation, competitive orientation, emotional orientation, business orientation, networking orientation, sustainability orientation, and micro-enterprise sustainability are evaluated and reported in Table 1. The reliability of the latent constructs was assessed with Cronbach’s alpha (CA), Dillon–Goldstein rho (DG rho), and composite reliability (CR) and reported. Consequently, CA values are good above the 0.70 benchmarks, and the least CR score achieved value (0.941) by the entrepreneurial orientation (Chin, 2010). Next, the DG rho must be above 0.70 to represent the appropriate reliability (Hair et al., 2019). The customer orientation achieved the bottommost score (0.942). The CR also needs to be above 0.70; the least score (0.953) realized by the entrepreneurial orientation for the current study (Chin, 2010). The convergent validity was accomplished with the average variance extracted (AVE) value above 0.50 for all latent constructs above the 0.50 threshold (Hair et al., 2019). Finally, multicollinearity issues were estimated with the variance inflation factors (VIF). The VIF value of each factor is less than 3.3, suggesting that no major collinearity/problem was present (Chin, 2010). The results are provided in Table 1.

**TABLE 1:** Reliability and validity.

Variables	Items	Mean	SD	CA	DG *rho*	CR	AVE	VIF
Entrepreneurial orientation	6	5.214	1.092	0.941	0.942	0.953	0.772	1.492
Customer orientation	12	5.438	0.863	0.948	0.950	0.955	0.641	2.448
Market orientation	10	5.073	0.919	0.959	0.960	0.964	0.729	2.101
Product innovation	7	5.488	1.003	0.960	0.961	0.967	0.807	1.000
Process innovation	7	5.391	1.096	0.957	0.959	0.965	0.795	1.000
Enterprise performance	7	4.989	0.973	0.961	0.964	0.968	0.813	–

*SD, standard deviation; CA, Cronbach’s alpha; DG rho, Dillon–Goldstein’s rho; CR, composite reliability; AVE, average variance extracted; VIF, variance inflation factors. Source: Author’s data analysis.*

The current study used the loading and cross-loading, Fornell–Larcker Criterion, and Hetro-trait and Mono-trait (HTMT) ratio to evaluate the discriminant validity (Hair et al., 2019). Discriminant validity for the current study was further verified *via* a comparison between the loadings and cross-loadings for the tested constructs. Generally, loadings contribute an item to the latent variable it belongs to Hair et al. (2019). In contrast, cross-loading is the contribution of an item to other latent variables (see Appendix Table 1). The Fornell–Larcker Criterion was appraised by taking the square root of the AVE of the construct, and the score must be greater than the corresponding correlation coefficient to establish the discriminant validity. This study constructs show suitable discriminant validity as depicted in Table 2. Next, the study’s HTMT ratio was utilized to evaluate the discriminant validity (Henseler et al., 2015). All the HTMT ratios were less than the 0.900 bounds and professed that the study latent constructs achieved appropriate discriminant validity (Hair et al., 2019). The results are provided in Table 2.

**TABLE 2:** Discriminant validity.

*Fornell–Larcker criterion*	MKO	CSO	ENO	PIN	PSI	ETP
Market orientation	*0.854*					
Customer orientation	0.721	*0.800*				
Entrepreneurial orientation	0.462	0.570	*0.879*			
Product innovation	0.592	0.648	0.726	*0.784*		
Process innovation	0.392	0.512	0.615	*0.295*	*0.495*	
Enterprise performance	0.378	0.362	0.382	0.338	0.508	*0.902*
***Heterotrait–Monotrait ratio (HTMT)***						
Market orientation	–					
Customer orientation	0.756	–				
Entrepreneurial orientation	0.484	0.595	–			
Product innovation	0.619	0.678	0.238	–		
Process innovation	0.398	0.510	0.615	0.560	–	
Enterprise performance	0.391	0.377	0.401	0.244	0.530	–

*MKO, market orientation; CSO, customer orientation; ENO, entrepreneurial orientation; PIN, product innovation; PSI, process innovation; ETP, enterprise performance.*

*The off-diagonal values in the Fornell–Larcker Criterion matrix are the correlations between the latent constructs, and diagonal are square values of AVEs.*

*Source: Author’s data analysis.*

### Path Analysis

Afterward, we analyze and report the path analysis; the *r*^2^ value shows that 53.1% of the total variation in product innovation is explained by market orientation, customer orientation, and entrepreneurial orientation. The *f*^2^ value of 0.405 indicates a strong effect of entrepreneurial orientation on product innovation. Finally, the *Q*^2^ value of 0.102, which is more than 0, indicates that the observed values are well reconstructed and that the model has medium predictive relevance (Hair et al., 2019). Next, the *r*^2^ value indicates that 45.9% of the sample’s total variation in process innovation can be explained by market orientation, customer orientation, and entrepreneurial orientation. Finally, the *Q*^2^ value of 0.227 indicates that the model has medium predictive relevance (Hair et al., 2019).

The *r*^2^ value suggests that product innovation explains 12.8% of the total variation in enterprise performance. The *f*^2^ value of 0.147 specifies a moderate effect of product innovation on enterprise performance. Finally, the *Q*^2^ value of 0.425 directs that the observed values are well reconstructed and that the model has high predictive relevance (Chin, 2010). The *r*^2^ value designates that 28.3% of the total variation in enterprise performance across the sample can be explained by process innovation. Finally, the *Q*^2^ value of 0.360 indicates that the observed values are well reconstructed and that the model has high predictive relevance (Hair et al., 2019).

Next, the path values reported that the effect of market orientation on product innovation is also positive; however, the *p-*value of 0.119 is not significant at the 5% level, indicating that the relationship is not statistically significant. It offers no support to accept the H1a. The entrepreneurial and customer orientation has significant (at the 5% level) positive effects on product innovation. The outcome suggests accepting the H2a and H3a. The results show that the market orientation suggestively impacts process innovation, and customer orientation significantly influences process innovation. Next, the entrepreneurial orientation is significantly instigated by process innovation among the examined manufacturing SMEs. The result suggests accepting H1b, H2b, and H3b. The result is depicted in Table 3.

**TABLE 3:** Product innovation model.

Hypo		Beta	T	*P*	CI–Min	CI–Max	*r* ^2^	*f* ^2^	Q^2^	Decision
***Product innovation***										
H1a	MKO → PIN	0.088	1.568	0.119	−0.022	0.201		0.008		Reject
H2a	CSO → PIN	0.208	2.982	0.002	0.068	0.345	0.531	0.038	0.102	Accept
H3a	ENO → PIN	0.533	11.427	0.000	0.446	0.619		0.405		Accept
***Process innovation***										
H1b	MKO → PSI	0.282	4.597	0.000	0.165	0.400		0.069		Accept
H2b	CSO → PSI	0.150	2.028	0.032	0.026	0.296	0.459	0.017	0.227	Accept
H3b	ENO → PSI	0.370	5.824	0.000	0.242	0.496		0.170		Accept
***Enterprise performance***										
H4a	PIN → ETP	0.358	7.225	0.000	0.263	0.445	0.135	0.147	0.425	Accept
H4b	PSI → ETP	0.532	13.824	0.000	0.448	0.607	0.283	0.394	0.360	Accept

*MKO, market orientation; CSO, customer orientation; ENO, entrepreneurial orientation; PIN, product innovation; PSI, process innovation; ETP, enterprise performance. Source: Author’s data analysis.*

The coefficient for product innovation shows a significant positive effect on enterprise performance. The result suggests accepting the H4a. Lastly, the process innovation path coefficient significantly influences enterprise performance, and the finding confirms to accept H4b.

### Mediational Analysis

As presented in Table 4, the product innovation negatively mediated the relationship between the market orientation and enterprise performance, and it offers no statistical support to accept the HM1a. The results show that the relationship statistically mediates product innovation between customer orientation and enterprise performance for the mediating effect of product innovation. The finding confirms to accept HM1b. Likewise, a significant (*p-*value < 0.05) mediational effect of product innovation exists between entrepreneurial orientation and enterprise performance across the study sample. The results suggest accepting HM1c.

**TABLE 4:** Mediational analysis.

*Mediating effect of product innovation*		Beta	T	*p*	CI–Min	CI–Max	Decision
HM1a	MKO → PIN → ETP	0.032	1.462	0.144	−0.008	0.077	No Mediation
HM1b	CSO → PIN → ETP	0.075	2.864	0.004	0.024	0.127	Mediation
HM1c	ENO → PIN → ETP	0.191	5.976	0.000	0.133	0.253	Mediation
***Mediating effect of product innovation***							
HM2a	MKO → PSI → ETP	0.150	4.358	0.000	0.087	0.218	Mediation
HM2b	CSO → PSI → ETP	0.080	2.139	0.033	0.014	0.158	Mediation
HM2c	ENO → PSI → ETP	0.197	5.383	0.000	0.128	0.259	Mediation

*MKO, market orientation; CSO, customer orientation; ENO, entrepreneurial orientation; PIN, product innovation; PSI, process innovation; ETP, enterprise performance. Source: Author’s data analysis.*

Finally, the mediational analysis illustrates that the relationship between market orientation, customer orientation, and entrepreneurial orientation on enterprise performance is significantly mediated by process innovation. The findings support accepting HM2a, HM2b, and HM2c.

## Discussion

Firms need sustainable performance to remain competitive and sustain in the highly competitive business environment; however, innovation is vital for superior performance for large or small enterprises. SMEs are considered less competitive as SMEs are unable to integrate innovation as a core strategic tool with the strategic orientation to become competitive (Wang et al., 2016). This study investigated the influence of process innovation and product-related innovation on enterprise performance from strategic orientations (market, customer, and entrepreneurial) among Malaysian manufacturing SMEs.

The findings revealed that market orientation has a positive but not statistically significant effect on product innovation for the study sample. The study offers no support to accept H1a. The current finding of the study opposes the outcome posted by Na et al. (2019) that firm-level marketing orientation harness the product-level innovation that instigates the firm performance. Similar findings were reported by Utami et al. (2022). The following hypothesis examined and established that the marketing orientation significantly influences the process innovation among SMEs. The finding received its support from the work of Kim (2017) that firms improved their processes to meet the market need and demands. The market orientation commands the internal firms’ process to address the obligatory or market-driven requirements (Behnam and Cagliano, 2019).

The following hypothesis examined the influence of customer orientation on product-level innovation, and the result depicted a significant and positive effect of customer orientation on product innovation. The finding achieved its support from the work of Wang et al. (2016) that customer orientation drives product-level innovation among manufacturing firms. The customers drive the firms’ business, and firms are obliged to follow customer needs and demands by having the right kind of product-level innovations (Girod and Whittington, 2017). Afterward, our analysis confirmed a substantial effect of customer orientation on process-level innovation. Our result accords with the outcome postulated by Khan and Khan (2019) that customer orientation instigates process-level innovation among Pakistani ICT firms. The changing demands of customers guide the firm to realign the business processes to remain competitive and sustainable (Tutar et al., 2015).

The succeeding premise inspected the impact of the entrepreneurial orientation on product-level innovation, and the consequence represented a significant and positive outcome of entrepreneurial orientation on product innovation. The current outcome realized its sustenance from Nasir et al. (2017) that entrepreneurial orientation internally motivates product-level modernization, leading to product innovation. The entrepreneurial mindset manipulates the firms’ business to attempt new attributes in the product or services to achieve superior performance. Subsequently, our investigation authorizes a significant effect of entrepreneurial orientation on the firms’ innovation in processes. The study result consensuses with the outcome suggested by Roxas et al. (2017) that entrepreneurial orientation prompts innovation in the firms’ processes among Pilipino firms. The entrepreneurial approach directs the firms to try new technologies and improve the business processes to achieve higher performance (Muangmee et al., 2021).

Lastly, product and process innovation are found to positively impact the firm’s performance and support accepting H4a and H4b. Hence, it is logical that overall innovation has a positive and statistically significant effect on firm performance when combined. Our current study findings agree with Wang et al. (2016) that the firm’s innovation in products and services harnesses the firm’s performance and empowers the firm to gain a competitive advantage in the highly competitive market conditions.

The mediating analysis significantly influences product innovation between the relationship of strategic orientation (customer and entrepreneurial) and the SMEs’ performance. The result offers substantial support to accept HM1b and HM1c. However, the insignificant mediating effects of product innovation exist between the market orientation and SMEs performance and offer no support to accept HM1a. Moreover, the mediating investigation reveals that the significant mediating influence of process innovation exists between the strategic orientation (market, customer, and entrepreneurial) on the SMEs’ performance. These outcomes suggest admitting HM2a, HM2b, and HM2c.

## Implications and Conclusion

This research provides empirical evidence on the impact of strategic orientation components (market, entrepreneurial, and customer orientation) on deconstructed and unified default forms of innovation (product and process) and firm performance among Malaysian manufacturing SMEs. According to the findings, market orientation has a minor impact on product innovation but has a large impact on process innovation. Entrepreneurial and customer orientation, on the other hand, greatly activate process and product innovation.

### Theoretical Implication

Theoretically, the study significantly contributes to the scarcity of empirical studies concerning the impact of strategic orientations by SMEs on product or process innovation and organizational performance. The study addresses the inadequacy of propositions and empirical explorations concerning strategic orientation and innovation at the product and process level (Siti et al., 2017), mainly from Malaysian SMEs’ perspectives. Hence, we uniquely contribute in terms of strategic orientation and innovation dimensions by focusing solely on the internal environment within a specific segment of firms (manufacturing SMEs) in the emerging economy. The study offers a better understanding of strategic orientations and the interplay of SMEs’ levels of innovation (product and process), allowing them to harness their performance. This work explores product and process-level innovation, instigating SMEs’ superior performance in novelty, meeting the firm’s customer requirements, and gaining competitiveness. Moreover, the study addresses the criticism raised by Nasir et al. (2017) regarding the direct association of any particular strategic orientation toward a firm’s performance by examining the indirect effects of three strategic orientation dimensions on a firm’s performance. According to the findings of the study, product and process innovation have a significant impact on SMEs performance in Malaysian manufacturing SMEs.

### Practical Implications

In terms of practical implications, the study’s outcomes highlight that market, entrepreneurial, and customer-related strategies could help improve SMEs performance. The results suggest that policymakers should formulate and implement effective policies and programs primarily aimed at enhancing market orientation (gaining, disseminating, and reacting to market-generated intelligence), customer orientation (consumer-centered approach), entrepreneurial orientation (exploring new market opportunities), and entrepreneurialism (exploring new market opportunities) to harness SMEs-level innovation in products and processes. It would further empower SMEs to sustain and achieve superior firm performance in the highly competitive marketplace. The result suggests that market orientation was weak among the Malaysian manufacturing SMEs and did not permit the SMEs to engage in product-level innovation. SMEs management needs to be more vigilant toward market dynamics and react to the market trendiness to rightly adjust the SMEs’ offerings to meet the changing market demands.

Moreover, the lack of market orientation may not lead to the SMEs’ performance through product innovation. SMEs management is actively engaged in digital transformation to improve processes and gain competitiveness through effectiveness. The strategic orientation facilitates the firm’s preparedness to use technology for product and process improvements. SMEs management must focus on developing an entrepreneurial mindset as a critical driving force to achieve product and process-level innovation. The product and process-level innovations were transmitted to the SMEs’ performance.

This study is associated with three relevant limitations. First, the work utilized three dimensions of the strategic orientation (marketing, customer, and entrepreneurial orientations) influencing the product and service-level innovation. Future studies may consider including other strategic orientations aspects of competition, emotional, business, and networking facilitating the SMEs to engage in product and service-level innovation. Second, the work takes a general sample of SMEs to comprehensively evaluate the influence of the strategic orientation on product and service-level innovation. Future study needs to take a sample from specific industry SMEs as the SMEs’ working environment and internal working vary based on the industry. It helps offer an industry-specific understanding of the SMEs working in the industry. Moreover, the external factors also significantly influence innovation and promote local and international performance. Lastly, the current work is based on the sample taken from Malaysia. The same model may be utilized in other geographic locations to establish the holistic empirical support for the study model and apply the SMEs’ strategic orientation infusing the product innovation harnessing the SMEs’ performance sustainably.

## Data Availability Statement

The raw data supporting the conclusions of this article will be made available by the authors, without undue reservation.

## Ethics Statement

Ethical review and approval was not required for the study on human participants in accordance with the local legislation and institutional requirements. The patients/participants provided their written informed consent to participate in this study.

## Author Contributions

AA, NH, and SF engaged in research design, data collection, analysis, and reporting. AS and NZ actively played a role in analysis and wrote up. ZM played a role in drafting and management of data. All authors contributed to the article and approved the submitted version.

## Conflict of Interest

The authors declare that the research was conducted in the absence of any commercial or financial relationships that could be construed as a potential conflict of interest.

## Publisher’s Note

All claims expressed in this article are solely those of the authors and do not necessarily represent those of their affiliated organizations, or those of the publisher, the editors and the reviewers. Any product that may be evaluated in this article, or claim that may be made by its manufacturer, is not guaranteed or endorsed by the publisher.

## Appendix

**TABLE A1:** Loadings and cross-loadings.

	MKO	CSO	ENO	PIN	PSI	ETP
Market orientation–Item 1	***0.796***	0.493	0.318	0.374	0.380	0.279
Market orientation–Item 2	***0.831***	0.539	0.394	0.446	0.435	0.337
Market orientation–Item 3	***0.857***	0.610	0.362	0.451	0.422	0.299
Market orientation–Item 4	***0.841***	0.610	0.407	0.383	0.501	0.363
Market orientation–Item 5	***0.864***	0.613	0.415	0.426	0.519	0.349
Market orientation–Item 6	***0.863***	0.648	0.408	0.386	0.505	0.350
Market orientation–Item 7	***0.891***	0.634	0.425	0.416	0.483	0.313
Market orientation–Item 8	***0.877***	0.642	0.389	0.397	0.473	0.262
Market orientation–Item 9	***0.859***	0.688	0.396	0.400	0.452	0.270
Market orientation–Item 10	***0.857***	0.667	0.415	0.411	0.553	0.390
Customer orientation–Item 1	0.565	***0.742***	0.330	0.352	0.390	0.256
Customer orientation–Item 2	0.614	***0.822***	0.389	0.413	0.369	0.250
Customer orientation–Item 3	0.637	***0.819***	0.394	0.410	0.422	0.315
Customer orientation–Item 4	0.624	***0.844***	0.427	0.406	0.415	0.279
Customer orientation–Item 5	0.637	***0.835***	0.473	0.481	0.455	0.281
Customer orientation–Item 6	0.513	***0.766***	0.412	0.419	0.491	0.324
Customer orientation–Item 7	0.637	***0.853***	0.448	0.442	0.454	0.310
Customer orientation–Item 8	0.376	***0.656***	0.428	0.472	0.407	0.205
Customer orientation–Item 9	0.554	***0.816***	0.508	0.536	0.479	0.296
Customer orientation–Item 10	0.591	***0.815***	0.507	0.487	0.508	0.350
Customer orientation–Item 11	0.588	***0.799***	0.539	0.490	0.480	0.321
Customer orientation–Item 12	0.584	***0.812***	0.543	0.524	0.472	0.264
Entrepreneurial orientation–Item 1	0.342	0.436	***0.816***	0.533	0.558	0.322
Entrepreneurial orientation–Item 2	0.397	0.479	***0.880***	0.592	0.429	0.315
Entrepreneurial orientation–Item 3	0.334	0.487	***0.886***	0.645	0.536	0.339
Entrepreneurial orientation–Item 4	0.408	0.528	***0.916***	0.634	0.476	0.308
Entrepreneurial orientation–Item 5	0.466	0.525	***0.862***	0.583	0.535	0.372
Entrepreneurial orientation–Item 6	0.484	0.545	***0.909***	0.651	0.522	0.354
Product innovation–Item 1	0.481	0.526	0.600	***0.859***	0.402	0.319
Product innovation–Item 2	0.389	0.486	0.634	***0.914***	0.469	0.317
Product innovation–Item 3	0.484	0.556	0.584	***0.886***	0.382	0.311
Product innovation–Item 4	0.389	0.489	0.592	***0.920***	0.512	0.324
Product innovation–Item 5	0.413	0.513	0.628	***0.932***	0.513	0.332
Product innovation–Item 6	0.470	0.552	0.649	***0.903***	0.505	0.312
Product innovation–Item 7	0.400	0.485	0.656	***0.871***	0.572	0.337
Process innovation–Item 1	0.536	0.538	0.485	0.405	***0.820***	0.427
Process innovation–Item 2	0.460	0.470	0.486	0.465	***0.879***	0.467
Process innovation–Item 3	0.560	0.526	0.507	0.479	***0.874***	0.500
Process innovation–Item 4	0.409	0.441	0.485	0.462	***0.893***	0.479
Process innovation–Item 5	0.492	0.495	0.546	0.474	***0.929***	0.493
Process innovation–Item 6	0.482	0.496	0.548	0.530	***0.922***	0.464
Process innovation–Item 7	0.538	0.543	0.564	0.527	***0.922***	0.486
Enterprise performance–Item 1	0.334	0.325	0.320	0.333	0.455	***0.909***
Enterprise performance–Item 2	0.352	0.344	0.351	0.338	0.476	***0.934***
Enterprise performance–Item 3	0.378	0.333	0.349	0.334	0.493	***0.933***
Enterprise performance–Item 4	0.349	0.344	0.357	0.342	0.517	***0.931***
Enterprise performance–Item 5	0.318	0.293	0.329	0.273	0.435	***0.848***
Enterprise performance–Item 6	0.343	0.343	0.371	0.333	0.512	***0.888***
Enterprise performance–Item 7	0.305	0.297	0.331	0.301	0.457	***0.862***

*MKO, market orientation; CSO, customer orientation; ENO, entrepreneurial orientation; PIN, product innovation; PSI, process innovation; ETP, enterprise performance. The Italic values in the matrix above are the item loadings, and others are cross-loadings.*

*Source: Author’s data analysis.*
